# Supplementary material for: In Utero Transfer of Adeno-Associated Viral Vectors Produces Long-Term Factor IX Levels in a Cynomolgus Macaque Model
Source: Mol Ther. 2017 Apr 24;25(8):1843–53. doi: 10.1016/j.ymthe.2017.04.003 (PMC5542637; doi:10.1016/j.ymthe.2017.04.003)
Supplement: Document S1. Tables S1 and S2 [file mmc1.pdf]

YMTHE, Volume *25*

Supplemental Information

In Utero Transfer of Adeno-Associated Viral Vectors Produces Long-Term Factor IX Levels in a Cynomolgus Macaque Model

Citra N.Z. Mattar, Irene Gil-Farina, Cecilia Rosales, Nuryanti Johana, Yvonne Yi Wan Tan, Jenny McIntosh, Christine Kaeppel, Simon N. Waddington, Arijit Biswas, Mahesh Choolani, Manfred Schmidt, Amit C. Nathwani, and Jerry K.Y. Chan

Table S1. AAV peptide sequences for T cell intracellular stimulation studies (SUPPLEMENTARY)

AAV8 peptide sequences for ICS

H-AGGGAPMADNNEGAD-OH
H-PMADNNEGADGVGSS-OH
H-NEGADGVGSSSGNWH-OH
H-GVGSSSGNWHCDSTW-OH
H-SGNWHCDSTWLGDRV-OH
H-CDSTWLGDRVITTST-OH
H-PQYGYLTLNNGSQAV-OH
H-LTLNNGSQAVGRSSF-OH
H-GSQAVGRSSFYCLEY-OH
H-GRSSFYCLEYFPSQM-OH
H-YCLEYFPSQMLRTGN-OH
H-FPSQMLRTGNNFQFT-OH
H-RNSLANPGIAMATHK-OH
H-NPGIAMATHKDDEER-OH
H-MATHKDDEERFFPSN-OH
H-DDEERFFPSNGILIF-OH
H-FFPSNGILIFGKQNA-OH
H-GILIFGKQNAARDNA-OH
H-MLTSEEEIKTTNPVA-OH
H-EEIKTTNPVATEEYG-OH
H-TNPVATEEYGIVADN-OH
H-TEEYGIVADNLQQQN-OH
H-IVADNLQQQNTAPQI-OH
H-LQQQNTAPQIGTVNS-OH

AAV5 peptide sequences for ICS

H-QGADGVGNASGDWHC-OH
H-VGNASGDWHCDSTWM-OH
H-GDWHCDSTWMGDRVV-OH
H-DSTWMGDRVVTKSTR-OH
H-GDRVVTKSTRTWVLP-OH
H-TKSTRTWVLPSYNNH-OH
H-NFEFTYNFEEVPFHS-OH
H-YNFEEVPFHSSFAPS-OH
H-VPFHSSFAPSQNLFK-OH
H-SFAPSQNLFKLANPL-OH
H-QNLFKLANPLVDQYL-OH
H-LANPLVDQYLYRFVS-OH
H-PQPNGMTNNLQGSNT-OH
H-MTNNLQGSNTYALEN-OH
H-QGSNTYALENTMIFN-OH
H-YALENTMIFNSQPAN-OH
H-TMIFNSQPANPGTTA-OH
H-SQPANPGTTATYLEG-OH
H-QYSTGQVTVEMEWEL-OH
H-QVTVEMEWELKKENS-OH
H-MEWELKKENSKRWNP-OH
H-KKENSKRWNPEIQYT-OH
H-KRWNPEIQYTNNYND-OH
H-EIQYTNNYNDPQFVD-OH

Table S2: Primers for quantitative and LAM-PCR (SUPPLEMENTARY)

Primer	Sequence (5´-3´)
Pf scAAV-LP1-hFIXco	GGAGAGGAGCAGAGGTTGTC
Pr scAAV-LP1-hFIXco	TGGTGGTGCCTGAAGCTGAG
Pf β-actin	TCCTGTGGCATCCACGAAA
Pf β-actin	CCACGTCACACTTCATGATGG
Linear PCR	GTCGAGTGGATGTTGGAGGTG
Expo 1 vector	AGCTCCAAGGTCAGCAGGCA
Expo 1 LCI	GACCCGGGAGATCTGAATTC
Expo 2	GTGTTTGCTGTTTGCTGCTTGC
Expo 2 LCII	GATCTGAATTCAGTGGCACAG
Library prep vector	AATGATACGGCGACCACCGAGATCTACACTCTTTCCCTACACGACGCTCTTCCGATCT(BC)GCCCATTTTAGGGGTCACGA
Library prep LCIII	CAAGCAGAAGACGGCATACGAGATCGGTCTCGGCATTCCTGCTGAACCGCTCTTCCGATCTAGTGGCACAGCAGTTAGG
