# Supplementary material for: In Utero Transfer of Adeno-Associated Viral Vectors Produces Long-Term Factor IX Levels in a Cynomolgus Macaque Model
Source: Mol Ther. 2017 Apr 24;25(8):1843–53. doi: 10.1016/j.ymthe.2017.04.003 (PMC5542637; doi:10.1016/j.ymthe.2017.04.003)
Supplement: Document S2. Article plus Supplemental Information [file mmc2.pdf]

Molecular Therapy
Original Article

In Utero Transfer of Adeno-Associated Viral Vectors Produces Long-Term Factor IX Levels in a Cynomolgus Macaque Model

Citra N.Z. Mattar,[1] Irene Gil-Farina,[2] Cecilia Rosales,[3] Nuryanti Johana,[4] Yvonne Yi Wan Tan,[4] Jenny McIntosh,[3] Christine Kaeppel,[2,8] Simon N. Waddington,[5,6] Arijit Biswas,[1] Mahesh Choolani,[1] Manfred Schmidt,[2] Amit C. Nathwani,[3] and Jerry K.Y. Chan[4,7]

[1]Obstetrics & Gynaecology, Yong Loo Lin School of Medicine, National University of Singapore, Singapore 119077, Singapore; [2]Department of Translational Oncology, German Cancer Research Center and National Center for Tumor Diseases, 69120 Heidelberg, Germany; [3]UCL Cancer Institute, University College London, London WC1E 6BT, United Kingdom; [4]Reproductive Medicine, K.K. Women's and Children's Hospital, Singapore 229899, Singapore; [5]Institute for Women's Health, University College London, London WC1E 6BT, United Kingdom; [6]MRC Antiviral Gene Therapy Research Unit, Faculty of Health Sciences, University of the Witwatersrand, Johannesburg 2000, South Africa; [7]Duke-NUS Medical School, Singapore 169857, Singapore

The safe correction of an inherited bleeding disorder in utero prior to the onset of organ damage is highly desirable. Here, we report long-term transgene expression over more than 6 years without toxicity following a single intrauterine gene transfer (IUGT) at 0.9G using recombinant adeno-associated vector (AAV)-human factor IX (hFIX) in the non-human primate model we have previously described. Four of six treated animals monitored for around 74 months expressed hFIX at therapeutic levels (3.9%–120.0%). Long-term expression was 6-fold higher in males and with AAV8 compared to AAV5, mediated almost completely at this stage by random genome-wide hepatic proviral integrations, with no evidence of hot-spots. Post-natal AAV challenge without immunosuppression was evaluated in two animals exhibiting chronic low transgene expression. The brief neutralizing immune reaction elicited had no adverse effect and, although expression was not improved at the dose administered, no clinical toxicity was observed. This long-term surveillance thus confirms the safety of late-gestation AAV-hFIX transfer and demonstrates that postnatal re-administration can be performed without immunosuppression, although it requires dose optimization for the desired expression. Nevertheless, eventual vector genotoxicity and the possibility of germline transmission will require life-long monitoring and further evaluation of the reproductive function of treated animals.

INTRODUCTION

In-utero molecular correction of a genetic disease offers an opportunity to completely avoid end-organ damage in conditions that manifest early in life. We have demonstrated that supra-physiological human factor IX (hFIX) expression during the perinatal period is achievable with adeno-associated vector (AAV)-mediated intrauterine gene transfer (IUGT) in late gestation, an approach which may arbitrate the risk of perinatally lethal events like intracranial hemorrhage from similar clotting factor deficiencies.[1–4] This strategy leverages on fetal cells being the better recipients for therapeutic vehicles, given their superior transduction and differentiation efficiencies, and may be used to treat genetic diseases diagnosed late in pregnancy in the immunologically mature fetus.[1,5–8] The key clinical challenges of AAV-IUGT concern the long-term safety in the treated fetus, namely (1) oncogenic potential of these clinical AAV vectors; (2) durability of therapeutic expression; and (3) risk of germ-line transmission of proviral DNA. The threat of insertional mutagenesis still presents one of the most daunting obstacles in the clinical translation of both prenatal and postnatal gene transfer. Although AAVs remain primarily episomal and are less likely to induce oncogenesis than oncoretroviruses and lentiviruses,[9,10] the low-frequency integration events found in adult, neonatal, and fetal animal models[1,11,12] raise the possibility of malignant transformation by disruption of tumor suppressor genes or activation of proto-oncogenes.[13] In fact, AAV-related hepatocellular genotoxicity reported in rodents[11,14,15] and the presence of wild-type AAV2 integration sites documented in biopsies from patients with hepatocellular carcinoma[16] highlight the need for a proper safety assessment. Moreover, because oncoretroviral-mediated oncogenesis has been linked to integration at sites of active gene transcription,[10] we anticipate that post-treatment oncogenic events are more likely during fetal life when genes are more actively transcribed than at any other developmental stage.[17]

Received 18 March 2017; accepted 3 April 2017;
http://dx.doi.org/10.1016/j.ymthe.2017.04.003.

[8]Present address: Eurofins Medigenomix GmbH, 85560 Ebersberg, Germany

Correspondence: Citra N.Z. Mattar, Obstetrics & Gynaecology, Yong Loo Lin School of Medicine, National University of Singapore, Singapore 119077, Singapore.
E-mail: citramattar@nus.edu.sgp
Correspondence: Jerry K.Y. Chan, Reproductive Medicine, K.K. Women's and Children's Hospital, Singapore 229899, Sinapore; Duke-NUS Medical School, Singapore 169857, Singapore.
E-mail: jerrychan@nus.edu.sg

This is an open access article under the CC BY license (http://creativecommons.org/licenses/by/4.0/).

Table 1. Offspring Biodata and Outcomes following IUGT

				hFIX, Normal Antigen (%) (± SD)		Postnatal Challenge			
ID (Sex)	IUGT Dose (vg/kg)	GA at Birth (Days)	Birth Weight (g)	Peak	Steady State	Age (Months)	Dose (vg)	hFIX at Last Time Point (%)	Longest Follow-up Period (Months)
8-002 (M)	1.5×10^{13}	143	275	656.5 (33.2)	109.6 (32.7)	Not done		100	11
8-006 (M)	1.4×10^{13}	148	290	566.3 (474.0)	35.6 (17.5)	Not done		24.8	51
8-007[a] (F)	1.4×10^{13}	147	280	4.8 (3.0)	0.4 (1.0)	30	AAV8, 3.98E+11		
						46	AAV5, 4.36E+11	0.0	62
5-002 (M)	1.5×10^{13}	143	260	122.6 (57.9)	16.9 (7.0)	Not done		10.9	71
5-006 (F)	1.9×10^{13}	145	205	51.4 (48.9)	3.0 (3.1)	32	AAV5, 4.82E+11	3.7	63
5-007 (F)	1.6×10^{13}	147	255	21.3 (5.8)	5.4 (3.5)	Not done		1.6	63

ID, identity number.
[a]Subject not included in the previous paper.[1]

Specific concerns arise from the current knowledge of AAV gene therapy of direct relevance to the clinical application of IUGT. We previously demonstrated that vector load and transgene expression diminish as the AAV-IUGT recipient undergoes rapid neonatal growth, which in the clinical context could lead to sub-therapeutic expression after the initial peak.[1,7] In this event, a postnatal vector boost would have to be considered if transgene expression is needed life-long. Sex-specific differences in the stability of hepatocyte AAV-transduction and transgene expression have been described in postnatally treated murine subjects, with males consistently outperforming females due to greater endogenous testosterone production and androgen receptor-binding sites around the transgene promoter.[18,19] This pattern was not observed in adult macaques because equivalent transduction and expression were observed in males and females with AAV-FVII delivery, leading us to originally conclude that these observations were murine specific.[20] Additionally, viral transmigration across the blood-gonadal barrier presents the possibility of germ-line transmission, of which the resulting reproductive toxicity, if any, should be defined.[21,22] The resolution of these critical questions is of urgent importance in the clinical translation of this promising strategy and can only be reliably addressed in a relevant preclinical non-human primate model.[23,24] Using the experimental paradigm of hemophilia B with its modest therapeutic goal of 1% hFIX activity, we present the longest follow-up in a macaque model ever reported and describe the variables that will be impactful in gene therapy applications.

RESULTS

Demographics

12 fetuses were injected at 0.9G with a single dose of AAV-FIX, and six infants survived; the causes of premature mortality were previously described[1] (Table 1). These animals received a mean dose of 1.6E+13 vector genomes (vg) (SD ± 0.19), had a mean birth weight of 260.8 ± 27.6 g and were monitored to an age of 53.5 ± 19.9 months. AAV8 recipients (n = 3) included two males and one female, and AAV5 recipients (n = 3) comprised two females and one male. Two recipients were selected for postnatal vector challenge to dropping or persistently low expression with a deliberately low dose of AAV due to safety concerns with higher doses.[25]

Transgene Levels, Vector Load, and Expression Efficacy following IUGT

In our previously reported 22-month follow-up, we observed a stable hFIX expression peaking 30–60 days after delivery, followed by a rapid decline to a stable plateau.[1] We have longitudinally monitored six animals; 8007 is reported here for the first time (Table 1). Peak expression was observed to occur within the first 60 days of delivery in most animals and ranged from 656.5 ± 33.2% in 8002 to 4.8 ± 3.0% in 8007, which occurred sporadically at 5 months in this animal. Mean steady-state expression from day 60 was 109.6 ± 32.7% in 8002 and 35.6 ± 17.5% in 8006 over 11 and 61 months of observation, respectively. 8007 had much lower mean hFIX levels of 0.4 ± 1.0% over 72 months. Peak expression was highest in 5002 at 122.6 ± 57.9% and lowest in 5007 at 21.3 ± 5.8%. Mean steady-state expression among AAV5 subjects was 16.9 ± 7.0% in 5002, 3.0 ± 3.1% in 5006, and 5.4 ± 3.5% in 5007 over 74–82 months of observation. This was maintained despite weight gain, which increased by almost 16-fold by 4 years of age (Figures 1A–1F). Overall mean expression was 3-fold higher among AAV8 infants than AAV5 infants over a period of 71 months at 31.6 ± 58.8% and 10.8 ± 9.2%, respectively ($p = 0.003$). This was significant both during the periods of peak (216.9 ± 138.6% versus 41.8 ± 20.8%, $p = 0.04$) and steady-state expression (19.4 ± 2.2% versus 9.0 ± 0.4%, $p < 0.0001$) in AAV8 and AAV5, respectively. However, 8007 showed hFIX levels just over detection limits at 0.4 ± 0.9% over 62 months (Figure 1C), whereas expression in 5006 fell to <1% around 15–20 months (Figure 1E). Both were eventually challenged postnatally with another dose of AAV (black arrows); 5006 levels fluctuated randomly, and expression was ∼1.3% at the time of the second injection. There was only mild transient increase in hFIX in 5006 (to 4.8% 2 months after re-injection) and no discernible improvement in 8007, even with a second challenge at 46 months using AAV5 to circumvent existing anti-AAV8 capsid-specific antibodies. hFIX levels at final assessment are shown in Table 1. Assessment of vector load post-challenge

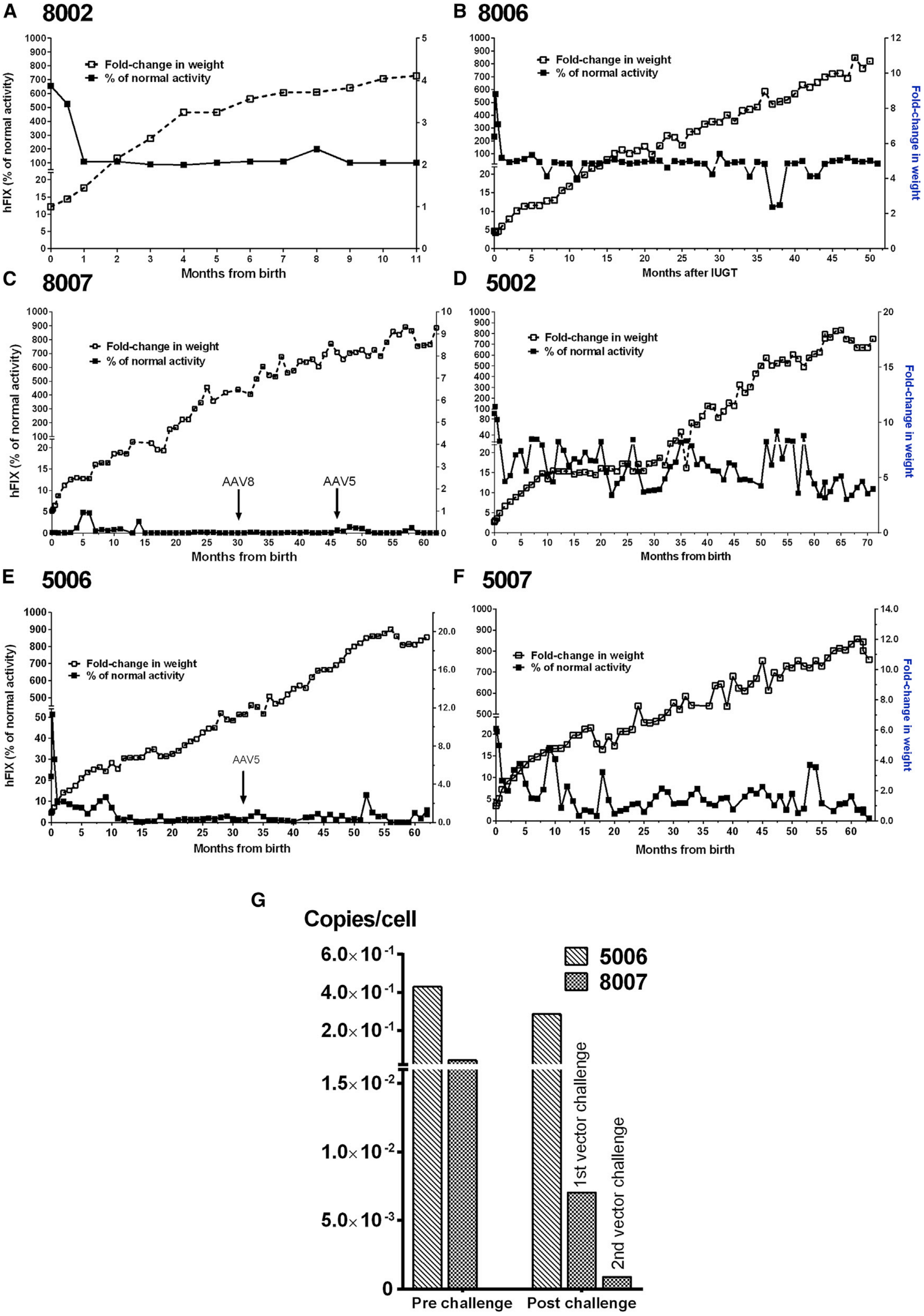

(legend on next page)

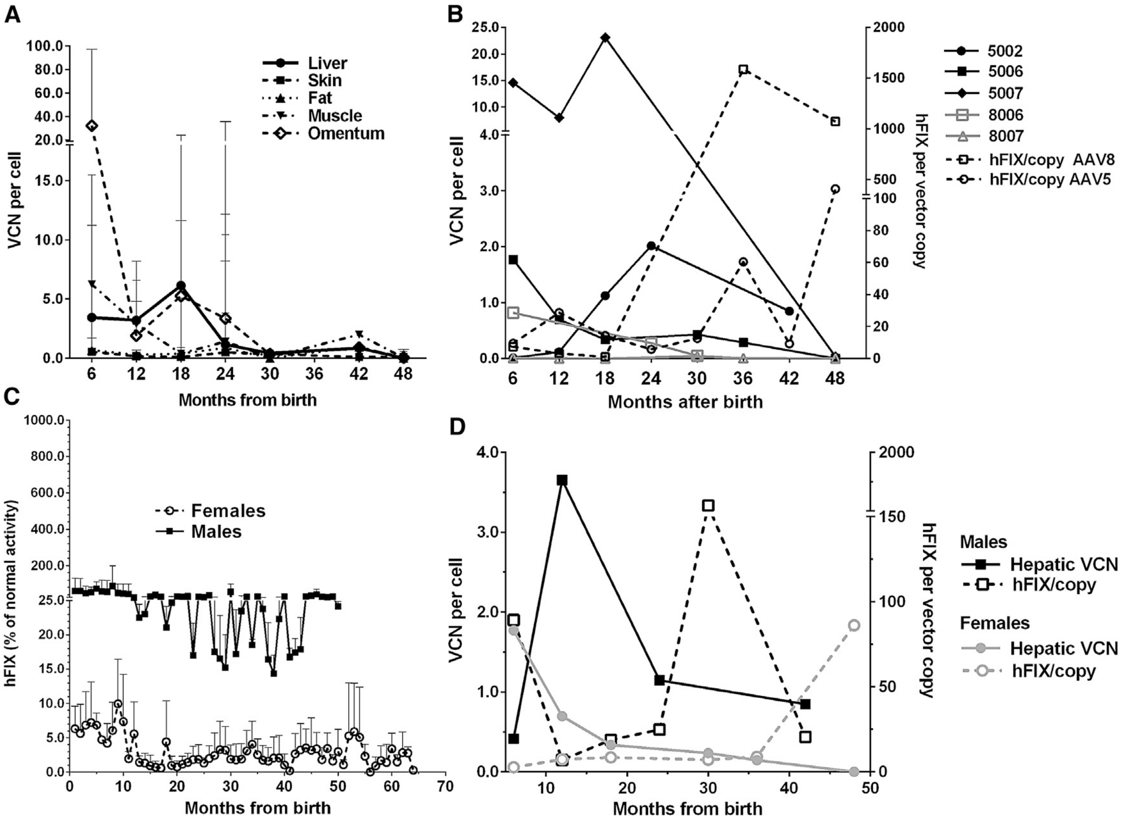

Figure 2. Efficacy of Transgene Expression

(A) Liver, skin, and peripheral fat showed stable low-level VCN between 6 and 48 postnatal months. VCN at 6 months was higher in muscle and omentum compared with the liver at 6 months and decreased rapidly thereafter. Vector copies persisted at stable low levels in the liver and other tissues in the presence of continued growth. (B) AAV8 recipients demonstrated more efficient expression (533.9 ± 748.4% hFIX activity per vector copy versus 68.6 ± 138.4% per vector copy with AAV5, ns). Comparison of individual hepatic VCN shows a 1 to 2 log-fold decrease in 5006 compared to other AAV5 recipients, whereas VCN in 8007 was lower than that in 8006 to the same extent. (C) Males had a log-fold higher hFIX expression than did females (38.9 ± 59.6% versus 3.6 ± 3.9%, $p < 0.0001$) throughout the surveillance period, (D) with a non-significant trend toward higher expression efficacy (210.1 ± 436.9% versus 20.1 ± 32.5% per vector copy in females). Error bars in (A) and (C) indicate SD.

demonstrated stable VCN in 5006 (~0.3 copies/cell) and a reduction from 0.04 to 0.001 copies/cell in 8007 post-challenge (Figure 1G). We did not analyze the functionality of hFIX protein through coagulation assays.

Hepatic vector copy number (VCN) in all organs had dropped between one and three log-folds by 18 postnatal months, with no statistical differences between them; mean hepatic VCN was 1.9 ± 2.1 copies/cell and omentum carried the highest load at 5.5 ± 11.0 copies/cell, whereas the vector load ranged from 0.2 to 1.6 copies/cell in skin, fat, and skeletal muscle (Figure 2A). Hepatic vector load was one log-fold higher in AAV8 than in AAV5 infants at all biopsy time points until around 3 years after birth, with 4.8 ± 4.9 copies/cell and 0.6 ± 0.4 copies/cell, respectively ($p = 0.03$, Figure 2B). Taking into account all biopsy time points over 48 months and the disproportionate distribution of males and females between serotype groups, AAV8 recipients demonstrated more efficient expression of hFIX (Figure 2B). For each hepatic vector copy, 533.9 ± 748.4% hFIX activity was achieved compared with 68.6 ± 138.4% activity per vector copy with AAV5 (not significant [ns]). Treated males had substantially higher hFIX levels regardless of AAV serotype used (Figure 2C). Overall expression was higher in males than in females (38.9 ± 59.6% versus 3.6 ± 3.9%, $p < 0.0001$), a trend seen during both peak (243.5 ± 151.7% versus 15.2 ± 7.3%, $p = 0.02$) and

Figure 1. Transgene Expression in IUGT Recipients

(A–F) hFIX levels peaked around 30–60 days postnatal and rapidly fell to a steadily maintained plateau level that was observed in most subjects despite the rapid growth during infancy; (A) 8002, (B) 8006, (C) 8007, (D) 5002, (E) 5006, (F) 5007. Expression was maintained at 31.6 ± 58.8% (AAV8) and 10.8 ± 9.2% (AAV5) over 11–71 months of observation, and was higher with AAV8 during both peak and steady-state periods. 5006 and 8007 exhibited suboptimal expression (<1%) and were challenged postnatally with another dose of AAV (arrows). (G) Despite this, there was only mild transient increase in 5006 and no discernible improvement in 8007. Assessment of vector load demonstrated stable VCN in 5006 (~0.3 copies/cell) and a reduction from 0.04 to 0.001 copies/cell in 8007 about 1 month post-challenge.

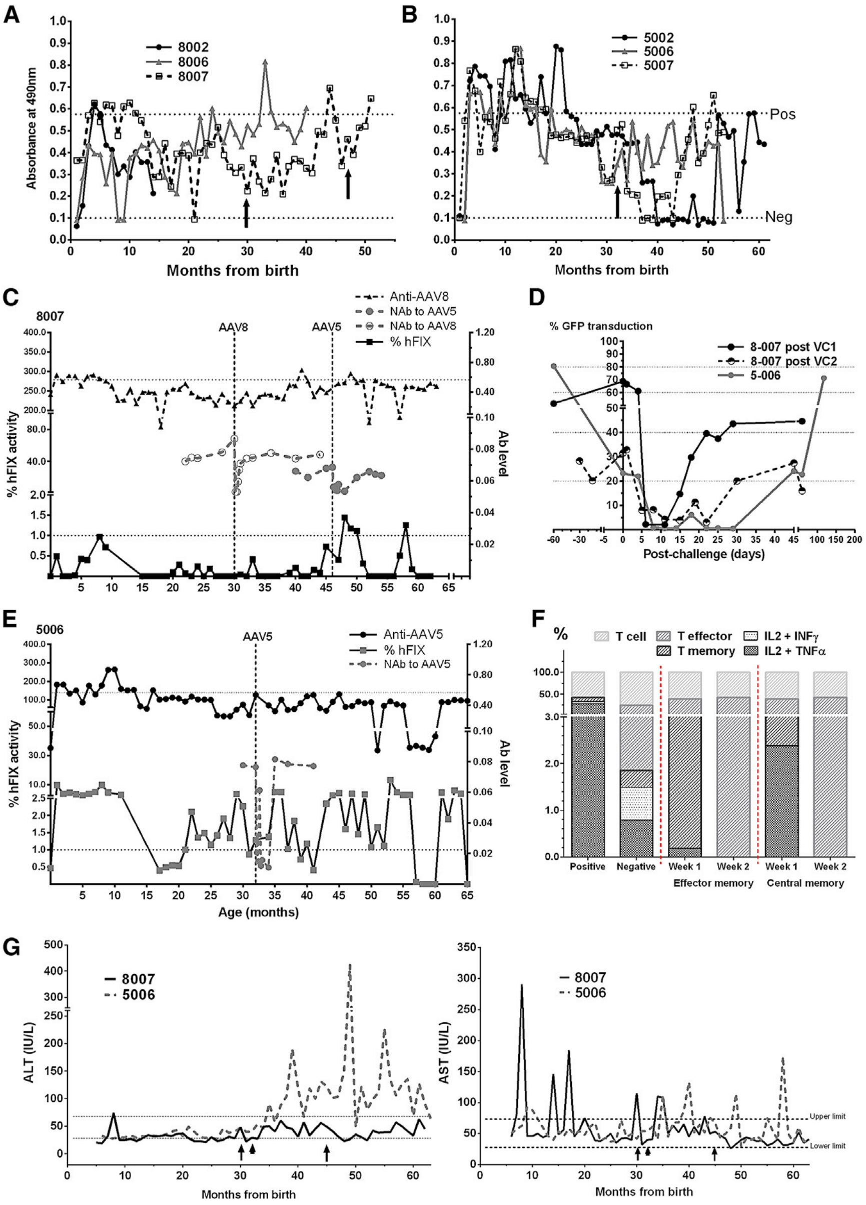

Figure 3. Immunogenicity of IUGT

(A) AAV8 recipients maintained low-level anti-AAV8 IgG after IUGT, which steadily increased following postnatal vector challenges in 8007 (arrows). Recipients of AAV5 mounted a more robust IgG response sustained for a year post-IUGT. (B) A single vector challenge to 5006 did not cause a substantial shift in IgG expression (arrow). (C) Expression in 8007 remained sub-therapeutic after both vector challenge (VC) (broken lines) at 30 and 46 months of age, peaking at 1.3% after the second challenge. Anti-AAV Ab levels remained subclinical. (D) NAbs were detected briefly by >50% loss of GFP expression in previously transduced cells in vitro. (E) Similar observations were made in 5006 in following VC (broken line) at 32 months; anti-AAV5 Abs remained stable. Upper dotted line in (C) and (E) indicates positive threshold for IgG response; lower dotted line indicates therapeutic minimum of 1% hFIX activity. (F) In 8007, IL-2 and TNF-α was expressed in 2% of CD8 central memory cells at week 1 and was no longer detectable by week 2 (positive ~23.5% IL-2, TNF-α, and INF-γ; negative ~1% expression). (G) 5006 showed intermittent increases in liver-specific ALT following VC (arrowhead), whereas minimal responses were observed in 8007 (arrows). Intermittent fluctuations in AST were observed before and after VC in both animals.

plateau phases (27.4 ± 15.7 versus 2.9 ± 2.0, $p < 0.0001$). Mean hepatic VCN was similar in males (2.8 ± 3.3 copies/cell) and females (1.2 ± 1.3 copies/cell over 48 months of surveillance, ns). This resulted in a non-significant trend toward a higher hFIX expression per vector copy in males than females (210.1 ± 436.9% versus 20.1 ± 32.5% per vector copy, Figure 2D).

Immunotoxicity and Genotoxicity

Next, we investigated the potential of AAV-IUGT to trigger an immune response. Besides the initial humoral reaction previously described,[1] continued surveillance showed that anti-AAV8-binding antibodies (Abs), initially peaking in the first 3–6 months, subsequently settled below the positive threshold for most of the remaining surveillance period (Figure 3A). Macaque 8007, exhibiting the lowest transgene expression, showed the most pronounced humoral response of AAV8 animals and maintained levels above the positive threshold for 7 months before resolution. Macaques 5002, 5006, and 5007 demonstrated a much more robust and prolonged anti-AAV5 reaction until 15 months, after which immunoglobulin G (IgG) expression dropped below the positive threshold for the remaining surveillance period (Figure 3B). Re-injected animals were checked for anti-AAV antibodies prior to postnatal treatment, which were found to be below the positive threshold (Figures 3A and 3B). Anti-AAV8 IgG in 8007 did not cross-react to AAV5. Expression in 8007 remained sub-therapeutic after vector challenges at 30 and 46 months of age, peaking at 1.4% after the second challenge. Prior to the first challenge, mean hFIX was 0.2 ± 0.3%; there was no change in expression with the first challenge of AAV8 (0.1 ± 0.2%) or with the second challenge of AAV5 (0.4 ± 0.5%, ns, Figure 3C). Anti-AAV-binding Ab levels remained subclinical, whereas neutralizing antibodies (NAbs) were detected for 20–30 days after challenges with both AAV8 and AAV5 by observing a dramatic drop in GFP-transduced 293T cells (to <50% baseline transduction), indicative of a brief neutralizing effect (Figures 3C and 3D). hFIX in 5006 increased briefly to 4.8% approximately 3 to 4 months post-challenge with AAV5, with another peak of 13.0% at 53 months of age, 21 months

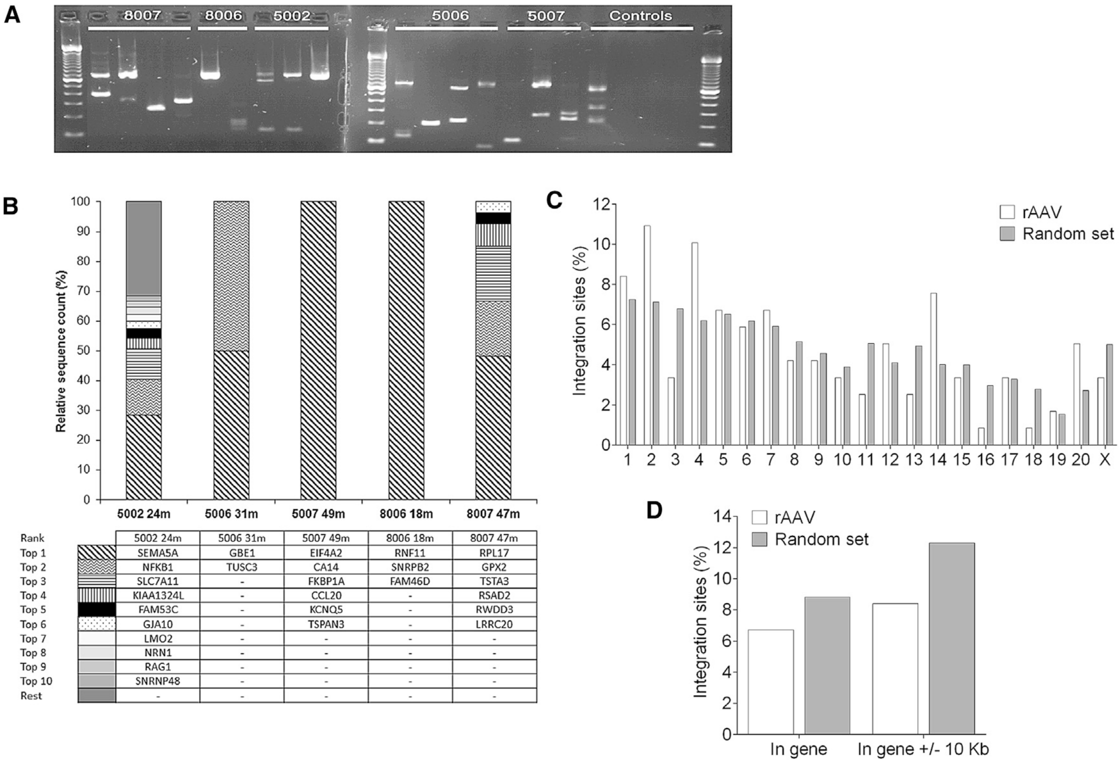

Figure 4. Vector Integration Analyses

(A) Gel electrophoresis of LAM-PCR products retrieved from the different samples and negative controls (untransduced genomic DNA and three water controls) using MseI for restriction digest. Broken lines indicate two separate gels in the same image. Despite the presence of bands in the untransduced DNA control, due to homologies between the vector and genomic sequences, no amplification was obtained in the sequencing library preparation, indicating that only AAV-derived amplicons were used in later analyses. Due to AAV concatemers, which are also amplified by LAM-PCR, not all bands correspond to ISs. (B) Relative sequence counts of the ten most prominent ISs retrieved were calculated in relation to all uniquely mappable IS sequences. The RefSeq identity of the gene located adjacent to or at the IS are listed. (C) Chromosomal distribution of retrieved ISs was analyzed and compared to a synthetic random dataset of 8,628 ISs to determine eventual integration hotspots. (D) Distribution of the ISs within gene coding and nearby regions in comparison to a random dataset.

post-challenge. Pre-challenge mean hFIX was 2.6 ± 2.4%, whereas post-challenge hFIX was 2.5 ± 2.6% (ns). Anti-AAV5 antibody levels remained stable, and NAbs were detected by day 7 and remained elevated for 35–40 days (Figures 3D and 3E). Anti-hFIX IgG expression was consistently negative throughout (data not shown). Evidence of cellular immune response to AAV capsid proteins were found in the first week after the first vector challenge in 8007, where central, but not effector, memory CD8 T cells demonstrated interleukin-2 (IL-2) and tumor necrosis factor α (TNFα) expression that was no longer detectable by the second week (Figure 3F). There were no CD4 responses from 8007, and neither CD4 nor CD8 T cells from 5006 expressed intracellular cytokines (data not shown). T cell analyses were not performed for 8007 following the second postnatal vector challenge (PVC). More fluctuations in alanine transaminase (ALT) and aspartate transaminase (AST) were observed in 5006 following vector challenge, whereas transaminases remained generally stable and unchanged in 8007 (Figure 3G). Despite this, no gross tumors or histological evidence of inflammation or mitotic events were detected at liver biopsy (performed between 6 and 49 months, data not shown).

To address genotoxic potential, we analyzed vector-genome junctions (integration sites [ISs]) on serial liver biopsies by linear amplification-mediated (LAM)-PCR (Figure 4A). 3,749,785 AAV-derived sequencing reads lead to the identification of 128 ISs, of which 119 were uniquely mappable to the macaque genome, whereas nine ISs were mappable to multiple loci (Table 2). Next, we analyzed the ten most prominent ISs through a semiquantitative estimation of the clonal size and found that no particular ISs were retrieved from consecutive biopsies (Figure 4B). One IS was found within the genotoxicity-associated *LMO2* gene in 5002, but was absent at later time points. No particular IS was repeatedly isolated from different animals. We observed no preferential integration within gene coding or nearby regions (Figure 4D) and no chromosomal integration hotspots when compared to a synthetic random dataset (Figure 4C). Although 8.4% of ISs were found to occur within cancer-related genes (included in the Cancer Gene Consortium, cBio, and retroviral-tagged cancer gene databases), this did not significantly differ from the 8.9% found in our synthetic random dataset. We compared the frequency of retrieved ISs (vector-genome) and concatemeric (vector-vector) junctions. The majority of retrieved sequences in

Table 2. IS Analysis in Sequential Liver Biopsies

		Hepatic Integration Sequences and Concatemers					
ID	Postnatal Challenge (Months)	Biopsy Time Points (Months)	Total IS	Mappable IS	IS/μg	IS (%)	Concatemers (%)
8006	–	18	427,064	3	2	100.0	0.0
8007	46	47	27	6	4	61.4	38.6
5002	–	24	608	111	74	87.4	12.6
5006	32	35	2	2	1.3	0.01	99.99
5007	–	49	550,809	6	4	100.0	0.0

Detailed summary of the IS retrieved by LAM-PCR. Total IS, number of retrieved sequences corresponding to vector-genome junctions; mappable IS, number of exactly mappable IS; % IS and concatemers, relative sequence count with respect to vector positive reads. ID, identity number.

pre- and non-challenged animals corresponded to IS (87.4%–100%), with only 0%–12.6% reflecting concatemers, suggesting that long-term transgene expression results primarily from integrated AAV genomes (Table 2). LAM-PCR analysis confirmed the re-appearance of vector concatemeric structures only in vector-challenged animals at similar time points, reflecting recent AAV transduction (38.6% concatemers versus 61.4% ISs in 8007 and 99.99% concatemers versus 0.01% ISs in 5006).

DISCUSSION

This is the longest reported follow-up of a clinically relevant macaque model for long-term outcomes of AAV-IUGT. Here, we demonstrate that a single AAV dose at 0.9G achieved durable and clinically relevant expression in 4/6 treated animals, and longitudinal surveillance confirms the overall absence of pathogenicity and genotoxicity in the treated offspring. The substantially higher expression in males and the contribution of AAV integration to long-term expression were novel and unexpected findings.

Males presented higher hFIX expression and hepatocyte transduction, and this sexual dimorphism in expression contrasts to studies on adult macaques.[20] These data should be interpreted with caution due to the small numbers of subjects and our limited ability to biopsy multiple lobes of the liver, which would have correlated better with hFIX expression. Although males were over-represented in the AAV8 group, both AAV8- and AAV5-treated females exhibited lower expression levels, indicating that prenatal diagnosis of fetal gender[26] and larger IUGT doses may be required to overcome this transduction barrier for other non-X-linked inherited diseases. This also suggests that the transduction barrier in females probably originates at the level of surface receptors controlling vector entry into the cell. Gender differences have also been reported in canines.[27] A large variation in hFIX levels was observed especially among AAV8-treated animals due to the exceptionally low expression in 8007 (female) and the supraphysiological expression in 8002 and 8006 (males). VCN among AAV8 animals was similar at each time point, and there was no notable maternal anti-AAV antibody production following IUGT (data not shown), as we have previously reported.[1] Compared to AAV8 recipients, AAV5 recipients showed lower hFIX expression and VCN, which may have resulted from the stronger and lengthier humoral response evoked. The temporal decline in hepatic VCN (up to three log-folds) was not in linear proportion to the increase in body weight, and we cannot currently explain this finding.

At the dose administered, IUGT recipients displayed similar expression kinetics as adult non-human primate (NHP), in which 1E+12 vg/kg of AAV-hFIX produced ~20% expression, peaking at 15 days with abrupt reversible immune-mediated loss.[28] Subsequent expression of anti-AAV activity was relatively subdued but may still have been high enough to block transduction from the second AAV dose. We previously reported baseline AAV integration of 10% from retrieved vector sequences acting in concert with episomal AAV to produce sustained transgene expression post-IUGT.[1] Nonetheless, current data reveal a progressive loss of vector episomes, due to liver growth, suggesting that sustained vector persistence and transgene expression is chiefly due to integrated vector forms. However, at late time points, both low VCN and episomal vector forms may have been the result of repeated sampling within the same hepatic area (allowing for fibrosis and regeneration) and thus are not reflective of non-biopsied areas. It would have been ideal to obtain random samples across the liver at each time point, but at greater physiological cost to the infants. Although still controversial, the genotoxic potential of AAV constitutes an unlikely but potentially major safety concern and requires lifelong surveillance. Despite the aforementioned high retrieval frequencies, ISs were distributed genome-wide, indicating the lack of preferential integration. One IS was found within the genotoxicity-associated *LMO2* gene in 5002 but was absent at later time points, and no further integration hotspots or single events were located within genes previously associated with AAV-driven liver cancer development. Accordingly, over 6 years post-treatment, no tumors were observed. These data suggest a low likelihood of malignant transformation and reassure us of its suitability for clinical use.

The 0.9G fetus is clearly immune competent and this influences long-term transgene expression. However, the sustained binding antibody expression following AAV-IUGT, with no loss of transgene expression in the majority of treated animals, suggests a larger contribution from non-neutralizing antibodies. The fetal immune system may be reactive but less efficient at clearing foreign antigens at this developmental stage, thus making late-gestation IUGT a useful intervention for genetic diagnoses made in advanced pregnancy.[29,30] Despite the

high dose of AAV8 used, only 8007's transgene expression did not peak early and did not reach our target of 1% hFIX during the first few months of life. The prolonged initial anti-AAV8 IgG response, the one- to two-log folds lower hepatic VCN (compared to males), and the transient spike in NAb and T cell cytokines suggest robust sensitization to AAV from first exposure. It is likely that the poor initial hepatocyte transduction caused consistently low transgene expression and failed to achieve liver-mediated tolerance, a key factor for sustained therapeutic expression.[31–33] The failure to increase hepatic transduction despite two PVC suggests the presence of specific unidentified inhibitors. Cross-reactivity of anti-AAV8 antibodies for AAV5 capsid proteins and vice versa is unlikely, and the use of an alternate serotype was, at this dose, insufficient to overcome this immune barrier.[34–36] Inflammation-mediated mechanisms inhibiting hFIX are also unlikely because ALT levels remained within normal limits before and after PVC. We did not do T cell assays serially, so we cannot correlate T cell responses to AAV with transaminases. AST was transiently increased in 5006 in the month following postnatal injection, with rapid resolution to baseline, whereas it remained at normal levels in 8007. Even with transient T cell activation, histological examination confirmed the absence of inflammation and hepatocyte destruction, suggesting that the mechanism of failed expression may differ from the inflammation-driven transgene loss found in adult humans that can be overcome with a short course of steroids.[25] Although transgene expression was not measurably improved in 5006 with PVC, the absence of activated T cells despite transient NAb expression and higher frequency of post-challenge concatemers suggest that repeated vector administration can be safely performed but requires optimization.

Naturally acquired NAbs to AAV5 and AAV8 usually accompany the development of high titers of anti-AAV2 NAbs, and their presence in children is usually transient.[37] Although our macaques not were screened for anti-AAV2 NAbs, this will be an important investigation in planning PVC for IUGT recipients, especially because the prevalence of acquired anti-AAV2 increases with age. In vitro assessment of neutralizing antibodies, cross-reacting IgG, and reactive T cells will be crucial when evaluating an IUGT recipient's suitability for postnatal vector re-administration, and future clinical use may also include immunomodulation to abrogate their effects.[28,38] The PVC dose here was kept to the lowest used in clinical trials in order to minimize adverse immune reactions.[25] In the absence of pre-existing inhibitors, we may expect a larger dose to overcome the transduction barrier and achieve therapeutic expression in under-expressing subjects.

Our study demonstrates the potential usefulness of the AAV-IUGT approach to hereditary genetic conditions, with potential early pathology due to its high efficacy and somewhat safe clinical and biochemical toxicity profile. It must be acknowledged that the observed long-term expression is mediated almost exclusively by AAV insertional mechanisms, and the potential for oncogenesis cannot be excluded yet. In life-threatening conditions or diseases conferring substantial morbidity presenting early in life, AAV-IUGT is safe and effective. For these reasons and other widely described advantages, even late-gestation gene transfer may be preferable to treatment in early childhood.[23] Precedents have been set with the early treatment of severe combined immunodeficiencies in young children, in whom the substantial benefits of stem cell gene therapy outweigh the risk of leukemogenesis.[39,40] Integration occurred more frequently than we anticipated from adult animal data, likely due to the highly open structure of the fetal genome.[41] In the practical context, a decision for AAV-IUGT will require lifelong surveillance for vector-related complications, including liver cancer. It is certainly reassuring that at least no hotspots were discovered. A clear distinction in expression efficacy is observed between males and females, which could mean that AAV-IUGT may be limited in its initial application to X-linked disorders or male fetuses. Although perhaps of limited prenatal use for bleeding disorders, AAV-IUGT can be also tailored—by optimizing serotype, gestational age, and dose—to a variety of life-threatening conditions, providing a promising early clinical intervention that may influence the development of personalized medicine.

MATERIALS AND METHODS

Animal Experiments, IUGT, and Surveillance

All procedures were performed in *Macaca fascicularis*, strictly adhering to recommendations from the Institutional Animal Care and Use Committee (IACUC) at the National University of Singapore and Singapore Health Services Pte (IACUC 2009-SHS-512). In vivo work was conducted at the SingHealth Experimental Medicine Centre (Singapore), accredited by the Association for Assessment and Accreditation of Laboratory Animal Care International (AAALAC). Fetal injections, together with prenatal and postnatal surveillance, have been described previously.[1,24] Briefly, macaque fetuses were injected with 4E+12 vg of scAAV-LP1-hFIX co-vector pseudotypes 8 or 5 at 0.9G based on pre-existing maternal seropositivity (Table 1).[34] Offspring were delivered surgically and monitored for tissue-specific hFIX expression and toxicity.[1,24] Serial biopsies of the liver were performed either by open midline laparotomy, during which additional samples of omentum, skin, subcutaneous fat, and skeletal muscle were obtained,[24] or with a minimally invasive approach by passing a Quick Core needle (Cook Medical) through a superficial skin incision into the liver under direct ultrasound guidance to obtain core biopsies. All procedures were performed under general anesthesia.

Molecular Analyses

Quantification of vector DNA content in serum and tissues was performed by qPCR as previously described using 100 nM of each primer[1] (Table S2). VCN was calculated per 6.6 pg of DNA in diploid cells. Expression of hFIX was determined by sandwich ELISA with anti-hFIX Capture Antibody (1:100 dilution).[34]

Generation of AAV8 and AAV5 Peptide Libraries for T Cell Intracellular Cell Staining

AAV8 (GenBank AF513852.1) and AAV5 (NCBI RefSeq NC_006152.1 and GenBank AF085716.1) capsid protein sequences obtained from NCBI were used to generate the AAV8 and AAV5

peptide pools, respectively.[42–44] Peptides generated from VP1, VP2, and VP3 capsid proteins of both serotypes were prepared as 15-mer overlapping by 10–12 amino acids (thinkpeptides, ProImmune). Peptides were resuspended in water at a stock concentration of 5–10 mg/mL, and pools were prepared at a final concentration of 2.5 μg/mL in PBS per peptide (Table S1).

Immunological Analyses

Humoral response: the presence of binding Abs reactive against the vector capsid (AAV) and transgene (hFIX) and anti-AAV NAbs were determined by semiquantitative sandwich ELISA and in-vitro inhibition assays of GFP transduction of 293T cells respectively, described previously.[1] The negative and positive thresholds of the semiquantitative anti-AAV ELISA were determined from the readouts of immunologically naive infant macaques not exposed to the vector and from adult macaques immunized with each vector.

Cell-mediated response: T cell activity was analyzed following vector challenge by intracellular cell staining (ICS) in a protocol adapted from Li and colleagues.[45] Peripheral blood mononuclear cells (PBMNCs) collected from IUGT recipients and naive adult NHP were isolated by density centrifugation using Ficoll-Paque and cryopreserved in DMSO (both Sigma-Aldrich) with fetal bovine serum (FBS) (GIBCO, Life Technologies) and Dulbecco's Modified Eagle's medium (GIBCO) in a ratio of 1:4:5 until analysis.[46] Cells were rapidly thawed, washed with sterile PBS, and incubated overnight in RPMI 1640 (Life Technologies) with 10% FBS and 1% penicillin-streptomycin (GIBCO) at 37°C in 5% CO_2. Treated NHP cells were washed with Hank's Balanced Salt Solution (Sigma-Aldrich) supplemented with 2 units/mL of DNase I (Thermo-Scientific), resuspended in RPMI 1640, and stimulated with the relevant AAV capsid peptide pool by 6-hr incubation in the presence of anti-CD28 (clone CD28.2), anti-CD49d (clone 9F10), and Brefeldin A. Naive NHP cells were fixed in 4% paraformaldehyde (PFA) for 10 min following overnight incubation and washing, centrifuged at 400 × *g* for 15 min, and resuspended in 1 mL of fluorescence-activated cell sorting (FACS) buffer (1% BSA in PBS).

To analyze AAV8 capsid-specific CD8+ and CD4+ T cells, the subject's stimulated PBMNC cells were stained with LIVE/DEAD Fixable Violet Dead Cell stain kit Pacific Blue (Invitrogen) and incubated with the following antibodies for 30 min in the dark at 4°C: anti-CD14-Pacific Blue (clone M5E2), anti-CD16-Pacific Blue (clone 3G8), anti-CD20-Pacific Blue (clone 2H7, AbD serotec), anti-CD8-APC-H7 (clone SK1), anti-CD4-Alexa700 (clone OKT4; eBioscience), anti-CD95-PE-Cy5 (clone DX2), anti-CD28-PE-Texas Red (clone CD28.2; Beckman Coulter), and anti-CCR7-PE (clone 150503; R&D Systems). Cells incubated without peptides were used as negative controls, whereas positive controls consisted of PBMNCs stimulated with phorbol 12-myristate 13-acetate (final concentration of 0.05 μg/mL) and ionomycin (final concentration of 5 μg/mL). Cells were permeabilized with Cytofix/Cytoperm for 20 min at room temperature. Intracellular staining was performed with anti-IFN-γ-APC, anti-IL-2-FITC, anti-TNF-α-PE-Cy7, and anti-CD3-PerCP-Cy5.5 for 30 min at 4°C. Cells were washed with Cytofix/Cytoperm once, fixed with 4% PFA, and then analyzed by FACS. Single-color controls were provided using CompBeads Anti-Mouse, Anti-Rat Ig kappa, and FITC single color CompBeads (BD Biosciences, unless stated). Live cells were singly gated to exclude CD14+, CD16+, and CD20+ and dead cells. Live CD3+ cells were gated for CD8+ and CD4+ individually, then gated for CD95 and CD28. Putative effector cells (CD95hiCD28hi) were analyzed for IL-2 and IFN-γ or IL-2 and TNF-α. Putative memory cells (CD95intCD28low) were gated onto CCR7. Central memory (TCM, CCR7hi) and effector memory (TEM, CCR7low) T-lymphocyte subsets were gated for IL-2, interferon γ (IFN-γ), and TNF-α. Positive samples were identified if at least 0.05% of the subpopulation showed staining for a particular cytokine.[45] Flow cytometry was performed using LSR Fortessa and FACSDiva software (BD Biosciences). Post-acquisition analyses were performed with Summit 4.2 (Beckman-Coulter).

Histology

Fixed liver biopsy specimens were stained with H&E[24] to assess for changes in cell architecture and nuclear atypia and for inflammatory cell infiltration.

ISs Analyses by LAM-PCR and Next-Generation Sequencing

LAM-PCR was performed[47] with primers listed in Table S2. Briefly, products from two linear-PCR amplification steps underwent restriction digest with MseI and MluCI for subsequent adaptor ligation. Two nested PCRs were then performed using vector- and adaptor-specific primers, and an additional PCR step allowed library preparation for MiSeq sequencing (Illumina). The resulting raw sequences were analyzed by automated bioinformatical tools for quality-filter, vector trimming, and identification of vector-genome (ISs) and vector-vector (concatemers) junctions. ISs were mapped to the macaque genome using University of California Santa Cruz (UCSC) BLAT tools and analyzed by automated data mining tools to characterize the vector's integration profile.[48]

Postnatal Vector Challenge

Female offspring 5006 and 8007 were subjected to one or two PVC, respectively (Figures 1C and 1E). Anti-capsid and anti-hFIX antibodies, liver transaminases, hematological indices, hepatic VCN, and liver histology were assessed just prior to PVC. Under general anesthesia (GA), animals received a slow peripheral intravenous (IV) injection and were monitored for 15 min during and after the infusion. Post-PVC hematological indices, transaminases, and hFIX levels were monitored bi-weekly and humoral and cell-mediated immune assays were monitored weekly for the first month. 3 months later, animals underwent ultrasound (US)-guided liver biopsy with Quick Core biopsy needles (Cook Medical) for analysis of VCN and inflammatory infiltrates.

Statistical Analyses

Results were analyzed using statistical software GraphPad Prism version 6.04 (GraphPad Software). Data are shown as mean (± SD) and analyzed using unpaired t tests with Welch's correction for

unequal SD; $p < 0.05$ was considered significant. Frequency of retrieval of integration sequences from tissue samples of subjects was compared by Fisher's exact test.

SUPPLEMENTAL INFORMATION

Supplemental Information includes two tables and can be found with this article online at http://dx.doi.org/10.1016/j.ymthe.2017.04.003.

AUTHOR CONTRIBUTIONS

C.N.Z.M. designed the animal and molecular experiments, analyzed samples, and wrote and revised the manuscript. N.J. and Y.Y.W.T. performed the animal experiments and analyzed samples. J.M. and C.R. produced the vector used in these experiments and analyzed samples. A.B. performed the animal experiments. M.S., I.G.-F., and C.K. performed vector integration analysis and revised the manuscript. M.C., A.C.N., and J.K.Y.C. designed the experiments and wrote and revised the manuscript.

CONFLICTS OF INTEREST

The authors declare no competing financial interests relevant to this paper. A.C.N. reports patents related to Factor IX in gene therapy vectors (U.S. Patent No. 8,030,065, U.S. Patent No. 8,168,425, and European Patent No. 1,804,839). M.S. is co-founder and chief executive officer of GeneWerk GmbH.

ACKNOWLEDGMENTS

This study funded by the Singapore's Ministry of Health's National Medical Research Council grant NMRC/CSA/043/2012. Salary support for C.N.Z.M. was provided by the Singapore's Ministry of Health's National Medical Research Council NMRC/TA/0003/2012 and NMRC/CSA-INV/0012/2016; M.C. received salary support from NMRC/CSA/007/2009 and NMRC/CSA/0059/2014; S.N.W. received funding from ERC (SOMABIO-260862), the NC3Rs (NC/L001780/1) and MRC grant MR/P026494/1; and J.K.Y.C. received salary support from NMRC/CSA/043/2012 and CSIRG/1383/2014. The authors thank Bryan Ogden, Darvi Michell B. Sergi, Jose Sebastian David, and Vivienne Liang for providing veterinary support in the care and upkeep of the macaque colony and Lay Geok Tan for assisting with sample collection.

REFERENCES

1. Mattar, C.N., Nathwani, A.C., Waddington, S.N., Dighe, N., Kaeppel, C., Nowrouzi, A., Mcintosh, J., Johana, N.B., Ogden, B., Fisk, N.M., et al. (2011). Stable human FIX expression after 0.9G intrauterine gene transfer of self-complementary adeno-associated viral vector 5 and 8 in macaques. Mol. Ther. *19*, 1950–1960.

2. Citak, A., Uçsel, R., Karaböcüoglu, M., Unüvar, A., and Uzel, N. (2001). A rare cause of intracranial hemorrhage: factor X deficiency. Pediatr. Emerg. Care *17*, 349–350.

3. Al-Allawi, N.A., Shamdeen, M.Y., and Rasheed, N.S. (2010). Homozygosity for the Mediterranean a-thalassemic deletion (hemoglobin Barts hydrops fetalis). Ann. Saudi Med. *30*, 153–155.

4. Chui, D.H. (2005). Alpha-thalassemia: Hb H disease and Hb Barts hydrops fetalis. Ann. N Y Acad. Sci. *1054*, 25–32.

5. Chan, J., O'Donoghue, K., de la Fuente, J., Roberts, I.A., Kumar, S., Morgan, J.E., and Fisk, N.M. (2005). Human fetal mesenchymal stem cells as vehicles for gene delivery. Stem Cells *23*, 93–102.

6. Frumento, G., Zheng, Y., Aubert, G., Raeiszadeh, M., Lansdorp, P.M., Moss, P., Lee, S.P., and Chen, F.E. (2013). Cord blood T cells retain early differentiation phenotype suitable for immunotherapy after TCR gene transfer to confer EBV specificity. Am. J. Transplant. *13*, 45–55.

7. Mattar, C.N., Waddington, S.N., Biswas, A., Johana, N., Ng, X.W., Fisk, A.S., Fisk, N.M., Tan, L.G., Rahim, A.A., Buckley, S.M., et al. (2013). Systemic delivery of scAAV9 in fetal macaques facilitates neuronal transduction of the central and peripheral nervous systems. Gene Ther. *20*, 69–83.

8. Dighe, N., Khoury, M., Mattar, C., Chong, M., Choolani, M., Chen, J., Antoniou, M.N., and Chan, J.K. (2014). Long-term reproducible expression in human fetal liver hematopoietic stem cells with a UCOE-based lentiviral vector. PLoS ONE *9*, e104805.

9. Condiotti, R., Goldenberg, D., Giladi, H., Schnitzer-Perlman, T., Waddington, S.N., Buckley, S.M., Heim, D., Cheung, W., Themis, M., Coutelle, C., et al. (2013). Transduction of fetal mice with a feline lentiviral vector induces liver tumors which exhibit an E2F activation signature. Mol. Ther. *22*, 59–68.

10. Nowrouzi, A., Cheung, W.T., Li, T., Zhang, X., Arens, A., Paruzynski, A., Waddington, S.N., Osejindu, E., Reja, S., von Kalle, C., et al. (2013). The fetal mouse is a sensitive genotoxicity model that exposes lentiviral-associated mutagenesis resulting in liver oncogenesis. Mol. Ther. *21*, 324–337.

11. Inagaki, K., Piao, C., Kotchey, N.M., Wu, X., and Nakai, H. (2008). Frequency and spectrum of genomic integration of recombinant adeno-associated virus serotype 8 vector in neonatal mouse liver. J. Virol. *82*, 9513–9524.

12. Kaeppel, C., Beattie, S.G., Fronza, R., van Logtenstein, R., Salmon, F., Schmidt, S., Wolf, S., Nowrouzi, A., Glimm, H., von Kalle, C., et al. (2013). A largely random AAV integration profile after LPLD gene therapy. Nat. Med. *19*, 889–891.

13. Rosas, L.E., Grieves, J.L., Zaraspe, K., La Perle, K.M., Fu, H., and McCarty, D.M. (2012). Patterns of scAAV vector insertion associated with oncogenic events in a mouse model for genotoxicity. Mol. Ther. *20*, 2098–2110.

14. Donsante, A., Miller, D.G., Li, Y., Vogler, C., Brunt, E.M., Russell, D.W., and Sands, M.S. (2007). AAV vector integration sites in mouse hepatocellular carcinoma. Science *317*, 477.

15. Chandler, R.J., LaFave, M.C., Varshney, G.K., Trivedi, N.S., Carrillo-Carrasco, N., Senac, J.S., Wu, W., Hoffmann, V., Elkahloun, A.G., Burgess, S.M., and Venditti, C.P. (2015). Vector design influences hepatic genotoxicity after adeno-associated virus gene therapy. J. Clin. Invest. *125*, 870–880.

16. Nault, J.C., Datta, S., Imbeaud, S., Franconi, A., Mallet, M., Couchy, G., Letouzé, E., Pilati, C., Verret, B., Blanc, J.F., et al. (2015). Recurrent AAV2-related insertional mutagenesis in human hepatocellular carcinomas. Nat. Genet. *47*, 1187–1193.

17. Li, T., Huang, J., Jiang, Y., Zeng, Y., He, F., Zhang, M.Q., Han, Z., and Zhang, X. (2009). Multi-stage analysis of gene expression and transcription regulation in C57/B6 mouse liver development. Genomics *93*, 235–242.

18. Davidoff, A.M., Ng, C.Y., Zhou, J., Spence, Y., and Nathwani, A.C. (2003). Sex significantly influences transduction of murine liver by recombinant adeno-associated viral vectors through an androgen-dependent pathway. Blood *102*, 480–488.

19. Li, Q., Ma, Y., Li, W., Xu, W., Ma, L., Fu, G., Tian, X., Wang, Y., Li, X., Bythwood, T., et al. (2014). A promoter that drives gene expression preferentially in male transgenic rats. Transgenic Res. *23*, 341–349.

20. Binny, C., McIntosh, J., Della Peruta, M., Kymalainen, H., Tuddenham, E.G., Buckley, S.M., Waddington, S.N., McVey, J.H., Spence, Y., Morton, C.L., et al. (2012). AAV-mediated gene transfer in the perinatal period results in expression of FVII at levels that protect against fatal spontaneous hemorrhage. Blood *119*, 957–966.

21. França, L.R., Auharek, S.A., Hess, R.A., Dufour, J.M., and Hinton, B.T. (2012). Blood-tissue barriers: morphofunctional and immunological aspects of the blood-testis and blood-epididymal barriers. Adv. Exp. Med. Biol. *763*, 237–259.

22. Siu, M.K., and Cheng, C.Y. (2012). The blood-follicle barrier (BFB) in disease and in ovarian function. Adv. Exp. Med. Biol. *763*, 186–192.

23. Mattar, C.N., Waddington, S.N., Biswas, A., Davidoff, A.M., Choolani, M., Chan, J.K., and Nathwani, A.C. (2012). The case for intrauterine gene therapy. Best Pract. Res. Clin. Obstet. Gynaecol. *26*, 697–709.

24. Mattar, C.N., Biswas, A., Choolani, M., and Chan, J.K. (2012). Animal models for prenatal gene therapy: the nonhuman primate model. Methods Mol. Biol. *891*, 249–271.

25. Nathwani, A.C., Tuddenham, E.G., Rangarajan, S., Rosales, C., McIntosh, J., Linch, D.C., Chowdary, P., Riddell, A., Pie, A.J., Harrington, C., et al. (2011). Adenovirus-associated virus vector-mediated gene transfer in hemophilia B. N. Engl. J. Med. *365*, 2357–2365.

26. Ho, S.S., Damayanti, Z., Chua, W.Y., Ng, B.L., Peh, C.M., Biswas, A., and Choolani, M. (2004). Non-invasive prenatal diagnosis of fetal gender using real-time polymerase chain reaction amplification of SRY in maternal plasma. Ann. Acad. Med. Singapore *33*, S61–S62.

27. Sarkar, R., Mucci, M., Addya, S., Tetreault, R., Bellinger, D.A., Nichols, T.C., and Kazazian, H.H., Jr. (2006). Long-term efficacy of adeno-associated virus serotypes 8 and 9 in hemophilia a dogs and mice. Hum. Gene Ther. *17*, 427–439.

28. Nathwani, A.C., Rosales, C., McIntosh, J., Rastegarlari, G., Nathwani, D., Raj, D., Nawathe, S., Waddington, S.N., Bronson, R., Jackson, S., et al. (2011). Long-term safety and efficacy following systemic administration of a self-complementary AAV vector encoding human FIX pseudotyped with serotype 5 and 8 capsid proteins. Mol. Ther. *19*, 876–885.

29. Hashimoto, D., Chow, A., Noizat, C., Teo, P., Beasley, M.B., Leboeuf, M., Becker, C.D., See, P., Price, J., Lucas, D., et al. (2013). Tissue-resident macrophages self-maintain locally throughout adult life with minimal contribution from circulating monocytes. Immunity *38*, 792–804.

30. Ginhoux, F., and Guilliams, M. (2016). Tissue-resident macrophage ontogeny and homeostasis. Immunity *44*, 439–449.

31. Cao, O., Dobrzynski, E., Wang, L., Nayak, S., Mingle, B., Terhorst, C., and Herzog, R.W. (2007). Induction and role of regulatory CD4+CD25+ T cells in tolerance to the transgene product following hepatic in vivo gene transfer. Blood *110*, 1132–1140.

32. Crudele, J.M., Finn, J.D., Siner, J.I., Martin, N.B., Niemeyer, G.P., Zhou, S., Mingozzi, F., Lothrop, C.D., Jr., and Arruda, V.R. (2015). AAV liver expression of FIX-Padua prevents and eradicates FIX inhibitor without increasing thrombogenicity in hemophilia B dogs and mice. Blood *125*, 1553–1561.

33. Sack, B.K., Merchant, S., Markusic, D.M., Nathwani, A.C., Davidoff, A.M., Byrne, B.J., and Herzog, R.W. (2012). Transient B cell depletion or improved transgene expression by codon optimization promote tolerance to factor VIII in gene therapy. PLoS ONE *7*, e37671.

34. Nathwani, A.C., Gray, J.T., McIntosh, J., Ng, C.Y., Zhou, J., Spence, Y., Cochrane, M., Gray, E., Tuddenham, E.G., and Davidoff, A.M. (2007). Safe and efficient transduction of the liver after peripheral vein infusion of self-complementary AAV vector results in stable therapeutic expression of human FIX in nonhuman primates. Blood *109*, 1414–1421.

35. Davidoff, A.M., Gray, J.T., Ng, C.Y., Zhang, Y., Zhou, J., Spence, Y., Bakar, Y., and Nathwani, A.C. (2005). Comparison of the ability of adeno-associated viral vectors pseudotyped with serotype 2, 5, and 8 capsid proteins to mediate efficient transduction of the liver in murine and nonhuman primate models. Mol. Ther. *11*, 875–888.

36. Nathwani, A.C., Reiss, U.M., Tuddenham, E.G., Rosales, C., Chowdary, P., McIntosh, J., Della Peruta, M., Lheriteau, E., Patel, N., Raj, D., et al. (2014). Long-term safety and efficacy of factor IX gene therapy in hemophilia B. N. Engl. J. Med. *371*, 1994–2004.

37. Li, C., Narkbunnam, N., Samulski, R.J., Asokan, A., Hu, G., Jacobson, L.J., Manco-Johnson, M.J., and Monahan, P.E.; Joint Outcome Study Investigators (2012). Neutralizing antibodies against adeno-associated virus examined prospectively in pediatric patients with hemophilia. Gene Ther. *19*, 288–294.

38. Mingozzi, F., Anguela, X.M., Pavani, G., Chen, Y., Davidson, R.J., Hui, D.J., Yazicioglu, M., Elkouby, L., Hinderer, C.J., Faella, A., et al. (2013). Overcoming pre-existing humoral immunity to AAV using capsid decoys. Sci. Transl. Med. *5*, 194ra92.

39. Gaspar, H.B., and Thrasher, A.J. (2005). Gene therapy for severe combined immunodeficiencies. Expert Opin. Biol. Ther. *5*, 1175–1182.

40. Howe, S.J., Mansour, M.R., Schwarzwaelder, K., Bartholomae, C., Hubank, M., Kempski, H., Brugman, M.H., Pike-Overzet, K., Chatters, S.J., de Ridder, D., et al. (2008). Insertional mutagenesis combined with acquired somatic mutations causes leukemogenesis following gene therapy of SCID-X1 patients. J. Clin. Invest. *118*, 3143–3150.

41. Manesia, J.K., Franch, M., Tabas-Madrid, D., Nogales-Cadenas, R., Vanwelden, T., Van Den Bosch, E., Xu, Z., Pascual-Montano, A., Khurana, S., and Verfaillie, C.M. (2016). Distinct molecular signature of murine fetal liver and adult hematopoietic stem cells identifies novel regulators of hematopoietic stem cell function. Stem Cells Dev., Published online February 13, 2017. http://dx.doi.org/10.1089/scd.2016.0294.

42. Chiorini, J.A., Kim, F., Yang, L., and Kotin, R.M. (1999). Cloning and characterization of adeno-associated virus type 5. J. Virol. *73*, 1309–1319.

43. Gao, G.P., Alvira, M.R., Wang, L., Calcedo, R., Johnston, J., and Wilson, J.M. (2002). Novel adeno-associated viruses from rhesus monkeys as vectors for human gene therapy. Proc. Natl. Acad. Sci. USA *99*, 11854–11859.

44. Sabatino, D.E., Mingozzi, F., Hui, D.J., Chen, H., Colosi, P., Ertl, H.C., and High, K.A. (2005). Identification of mouse AAV capsid-specific CD8+ T cell epitopes. Mol. Ther. *12*, 1023–1033.

45. Li, H., Lasaro, M.O., Jia, B., Lin, S.W., Haut, L.H., High, K.A., and Ertl, H.C. (2011). Capsid-specific T-cell responses to natural infections with adeno-associated viruses in humans differ from those of nonhuman primates. Mol. Ther. *19*, 2021–2030.

46. Nilsson, C., Aboud, S., Karlén, K., Hejdeman, B., Urassa, W., and Biberfeld, G. (2008). Optimal blood mononuclear cell isolation procedures for gamma interferon enzyme-linked immunospot testing of healthy Swedish and Tanzanian subjects. Clin. Vaccine Immunol. *15*, 585–589.

47. Schmidt, M., Schwarzwaelder, K., Bartholomae, C., Zaoui, K., Ball, C., Pilz, I., Braun, S., Glimm, H., and von Kalle, C. (2007). High-resolution insertion-site analysis by linear amplification-mediated PCR (LAM-PCR). Nat. Methods *4*, 1051–1057.

48. Arens, A., Appelt, J.U., Bartholomae, C.C., Gabriel, R., Paruzynski, A., Gustafson, D., Cartier, N., Aubourg, P., Deichmann, A., Glimm, H., et al. (2012). Bioinformatic clonality analysis of next-generation sequencing-derived viral vector integration sites. Hum. Gene Ther. Methods *23*, 111–118.

YMTHE, Volume *25*

Supplemental Information

In Utero Transfer of Adeno-Associated Viral Vectors Produces Long-Term Factor IX Levels in a Cynomolgus Macaque Model

Citra N.Z. Mattar, Irene Gil-Farina, Cecilia Rosales, Nuryanti Johana, Yvonne Yi Wan Tan, Jenny McIntosh, Christine Kaeppel, Simon N. Waddington, Arijit Biswas, Mahesh Choolani, Manfred Schmidt, Amit C. Nathwani, and Jerry K.Y. Chan

Table S1. AAV peptide sequences for T cell intracellular stimulation studies (SUPPLEMENTARY)

AAV8 peptide sequences for ICS	AAV5 peptide sequences for ICS
H-AGGGAPMADNNEGAD-OH	H-QGADGVGNASGDWHC-OH
H-PMADNNEGADGVGSS-OH	H-VGNASGDWHCDSTWM-OH
H-NEGADGVGSSSGNWH-OH	H-GDWHCDSTWMGDRVV-OH
H-GVGSSSGNWHCDSTW-OH	H-DSTWMGDRVVTKSTR-OH
H-SGNWHCDSTWLGDRV-OH	H-GDRVVTKSTRTWVLP-OH
H-CDSTWLGDRVITTST-OH	H-TKSTRTWVLPSYNNH-OH
H-PQYGYLTLNNGSQAV-OH	H-NFEFTYNFEEVPFHS-OH
H-LTLNNGSQAVGRSSF-OH	H-YNFEEVPFHSSFAPS-OH
H-GSQAVGRSSFYCLEY-OH	H-VPFHSSFAPSQNLFK-OH
H-GRSSFYCLEYFPSQM-OH	H-SFAPSQNLFKLANPL-OH
H-YCLEYFPSQMLRTGN-OH	H-QNLFKLANPLVDQYL-OH
H-FPSQMLRTGNNFQFT-OH	H-LANPLVDQYLYRFVS-OH
H-RNSLANPGIAMATHK-OH	H-PQPNGMTNNLQGSNT-OH
H-NPGIAMATHKDDEER-OH	H-MTNNLQGSNTYALEN-OH
H-MATHKDDEERFFPSN-OH	H-QGSNTYALENTMIFN-OH
H-DDEERFFPSNGILIF-OH	H-YALENTMIFNSQPAN-OH
H-FFPSNGILIFGKQNA-OH	H-TMIFNSQPANPGTTA-OH
H-GILIFGKQNAARDNA-OH	H-SQPANPGTTATYLEG-OH
H-MLTSEEEIKTTNPVA-OH	H-QYSTGQVTVEMEWEL-OH
H-EEIKTTNPVATEEYG-OH	H-QVTVEMEWELKKENS-OH
H-TNPVATEEYGIVADN-OH	H-MEWELKKENSKRWNP-OH
H-TEEYGIVADNLQQQN-OH	H-KKENSKRWNPEIQYT-OH
H-IVADNLQQQNTAPQI-OH	H-KRWNPEIQYTNNYND-OH
H-LQQQNTAPQIGTVNS-OH	H-EIQYTNNYNDPQFVD-OH

Table S2: Primers for quantitative and LAM-PCR (SUPPLEMENTARY)

Primer	Sequence (5´-3´)
Pf scAAV-LP1-hFIXco	GGAGAGGAGCAGAGGTTGTC
Pr scAAV-LP1-hFIXco	TGGTGGTGCCTGAAGCTGAG
Pf β-actin	TCCTGTGGCATCCACGAAA
Pf β-actin	CCACGTCACACTTCATGATGG
Linear PCR	GTCGAGTGGATGTTGGAGGTG
Expo 1 vector	AGCTCCAAGGTCAGCAGGCA
Expo 1 LCI	GACCCGGGAGATCTGAATTC
Expo 2	GTGTTTGCTGTTTGCTGCTTGC
Expo 2 LCII	GATCTGAATTCAGTGGCACAG
Library prep vector	AATGATACGGCGACCACCGAGATCTACACTCTTTCCCTACACGACGCTCTTCCGATCT(BC)GCCCATTTTAGGGGTCACGA
Library prep LCIII	CAAGCAGAAGACGGCATACGAGATCGGTCTCGGCATTCCTGCTGAACCGCTCTTCCGATCTAGTGGCACAGCAGTTAGG
